# The Impact of Access to Clinical Guidelines on LLM‐Based Treatment Recommendations for Chronic Hepatitis B

**DOI:** 10.1111/liv.70324

**Published:** 2025-09-02

**Authors:** Robert Siepmann, Carolin Victoria Schneider, Marc Sebastian von der Stueck, Iakovos Amygdalos, Karsten Große, Kai Markus Schneider, Maike Rebecca Pollmanns, Mohamad Murad, Joel Joy, Elena Kabak, Marcella Ricardis May, Jan Clusmann, Christiane Kuhl, Sven Nebelung, Jakob Nikolas Kather, Daniel Truhn

**Affiliations:** ^1^ Department of Diagnostic and Interventional Radiology University Hospital RWTH Aachen Aachen Germany; ^2^ Department of Gastroenterology, Metabolic Diseases and Intensive Care University Hospital RWTH Aachen Aachen Germany; ^3^ Department of Surgery and Transplantation University Hospital RWTH Aachen Aachen Germany; ^4^ Else Kroener Fresenius Center for Digital Health, Medical Faculty Carl Gustav Carus Technical University Dresden Dresden Germany; ^5^ Medical Oncology, National Center for Tumor Diseases (NCT) University Hospital Heidelberg Heidelberg Germany; ^6^ Department of Medicine 1 University Hospital and Faculty of Medicine Carl Gustav Carus, Technische Universität Dresden Dresden Germany

**Keywords:** chronic hepatitis B, GPT, guideline coherence, LLM

## Abstract

**Background and Aims:**

Large language models (LLMs) can potentially support clinicians in their daily routine by providing easy access to information. Yet, they are plagued by stating incorrect facts and hallucinating when queried. Increasing the context by providing external databases while prompting LLMs may decrease the risk of misinformation. This study compares the influence of increased context on the coherence of LLM‐based treatment recommendations with the recently updated WHO guidelines for the treatment of chronic hepatitis B (CHB).

**Methods:**

GPT‐4 was queried with five clinical case vignettes in two configurations: with and without additional context. The clinical vignettes were explicitly constructed so that treatment recommendations differed between the formerly applicable 2015 WHO guidelines and the updated 2024 ones. GPT‐4 with context was provided access to the updated guidelines, while GPT‐4 without context had to rely on its internal knowledge. GPT‐4 was accessed only a few days after the release of the new WHO guidelines. Treatment recommendations were compared regarding guideline coherence, information inclusion, textual errors, wording clarity and preciseness by seven physicians.

**Results:**

Using GPT‐4 with context increased the coherence of the treatment recommendations with the new 2024 guidelines from 51% to 91% compared to GPT‐4 without context. Similar trends were observed for all other categories, leading to an increase of 54% in preciseness and clarity, 24% in completeness of incorporating the case vignette information, and 12% in textual correctness.

**Conclusions:**

If LLMs are consulted by clinicians for medical advice, they should be given access to external data sources to increase the chance of providing factually correct advice.

**Summary**

- Large Language Models without access to external sources give incorrect treatment recommendations for hepatitis B that are not up‐to‐date.
- Provision of external databases improves clarity and coherence with up‐to‐date guidelines in patients with chronic hepatitis B.

**Abbreviations**
CHB: Chronic hepatitis B
HBV: Hepatitis B virus
LLM: Large language model
RAG: Retrieval augmented generation
WHO: World Health Organization

## 1 Introduction

The introduction of large language models (LLMs) such as OpenAI's GPT‐4, has ushered in a new era of possibilities in various fields, including healthcare [1, 2]. In this context, LLMs show not only potential for improving medical education but may also improve clinical decision‐making, enhance patient communication and speed up disease diagnosis [3]. However, cutting‐edge technology often comes with high expectations of productivity gains, which may not be directly realised in the initial period after implementation [4]. Against this background, LLMs are still in an early stage of development and have not yet overcome this so‐called ‘productivity paradox’ [5]. Besides different types of biases, LLMs often fall short of expectations regarding factual correctness as indicated by false statements that are not coherent with scientific facts [6], so‐called hallucinations. In this context, users often expect LLMs to function as a knowledge database. However, LLMs’ most interesting properties lie in the ability to perform reasoning based on data [7].

A promising use case of LLMs in health care is providing treatment recommendations for individual patients based on acknowledged guidelines [8]. However, there are several challenges that have to be addressed before LLMs can be incorporated into the clinical routine, one being high heterogeneity of the accuracy of LLM‐based treatment recommendations among studies, for example, ranging from 6.4% to 91.4% in digestive diseases [9]. One contributing issue is the lack of an acknowledged definition of accuracy and missing standardised rules in evaluations of LLMs in medical settings [9, 10]. To improve accuracy, LLMs should be provided with external context, such as guideline documents or knowledge databases [11]. This allows them to supplement their training data with up‐to‐date, relevant information when generating responses [9, 12]. Even more importantly, LLMs have a cut‐off date for their training data, so incorporating novel medical information published after that cut‐off date requires dedicated access to that information.

In the field of hepatology, first applications of LLMs for clinical use have already shown promising results, for example, when consulting GPT‐3.5 regarding the treatment of nonalcoholic fatty liver disease [13]. Another study developed a liver‐specific language model that integrated information from professional society guidelines during response generation, demonstrating higher clinical accuracy than GPT‐4 [14].

To further explore the role of incorporating context in hepatology applications of LLMs, this study evaluates the feasibility of GPT‐4 in guiding further management in chronic hepatitis B (CHB). We specifically chose this use case, since new guidelines for the prevention, diagnosis, care and treatment of people with chronic hepatitis B infection have been published recently (29th March 2024) [15] after the cut‐off date of training for GPT‐4. These guidelines replace the previous version published in 2015 [16]. The management of CHB involves complex decision‐making to prevent progression to advanced liver disease, necessitating personalised treatment plans aligned with evolving global guidelines. The new guidelines aim for more straightforward treatment recommendations for both adults and adolescents, increased eligibility for antiviral prophylaxis in pregnant women to prevent mother‐to‐child transmission of Hepatitis B virus (HBV), and enhancements in HBV diagnostics [15]. This increases treatment eligibility, especially where healthcare resources are limited. Due to the recency of the newly published guidelines and the resulting high probability that GPT‐4 had not yet been trained on them as well as the inherent complexity of CHB management, this setting is particularly suitable for assessing LLM‐based treatment recommendations depending on additional context. This allows not only for the evaluation of the benefit of adding contextual information, but also for revealing the limitations of out‐of‐the‐box LLMs when applied to rapidly evolving clinical topics. Therefore, the aim of this study is to investigate the influence of additional contextual information by means of prompt engineering on treatment recommendations given by a state‐of‐the‐art LLM (GPT‐4) in managing patients with CHB.

## 2 Material Und Methods

### 2.1 Study Design and Case Description

The study was conducted in accordance with the ethical principles outlined in the Declaration of Helsinki. As this study involved only hypothetical cases without the use of real patient data and did not include human subjects or animal experiments, ethical approval was not required.

Five case vignettes of clinically relevant scenarios of patients with chronic hepatitis B and an associated question concerning treatment recommendations were constructed by an internal medicine resident (C.V.S.) with 4 years of work experience and a special focus on hepatology (15 HBV patients treated per year on average), especially in research. The design process of the cases took place as follows: The policy brief of the World Health Organization (WHO) [15] was used to identify specific topics in which treatment recommendations have changed between the 2015 and 2024 ‘Guidelines for the prevention, care and treatment of persons with chronic hepatitis B infection’ by the WHO (2015: https://www.who.int/publications/i/item/9789241549059; 2024: https://www.who.int/publications/i/item/9789240090903). Subsequently, five case vignettes were constructed that fall within the scope of these topics. Two cases relate to ‘expanded eligibility for treatment’ (one for adolescents and one for adults) and one case refers to ‘expanding access to antiviral prophylaxis for prevention of mother‐to‐child transmission’, ‘alternative antiviral regimens for treatment’ and ‘point‐of‐care and reflex HBV DNA testing’, respectively. In every case, a factitious patient was constructed matching the eligibility criteria for the treatment recommendation of the respective topic (e.g., in terms of age, imaging findings, laboratory results, medical environment, comorbidities, etc.). All cases do not contain any personal health information. All cases were reviewed and approved by two physicians board‐certified in internal medicine (J.N.K. and K.G.) by ensuring that the factitious patients were representative of actual patient populations, including demographic details, laboratory results, imaging findings, and medical histories. Additionally, the respective treatment questions were reviewed with regard to clarity, clinical relevance and appropriate consideration of the WHO guideline changes.

Table 1 details the cases and their respective old and updated treatment recommendations. While the 2015 WHO guidelines informed the selection of topics for case vignette design, the 2024 WHO guidelines served as the sole gold standard for evaluating the correctness and coherence of all treatment recommendations (both with and without context).

**TABLE 1:** Case vignettes and the corresponding treatment recommendations by WHO guidelines.

Case number	Case description	Question	Treatment recommendations based on 2024 WHO guidelines
1	A 14 year old female patient is diagnosed with chronic hepatitis B by testing positive for HBsAg. The patient has no cirrhosis, but significant fibrosis diagnosed by transient elastography	According to the ‘Guidelines for the prevention, diagnosis, care and treatment for people with chronic hepatitis B infection’ by the WHO, should this patient be treated?	‘Treatment is recommended for all adults and adolescents (2015: adults only) […] with chronic hepatitis B […] with: Evidence of significant fibrosis (2015: only cirrhosis) (≥F2b) based on an APRI score of > 0.5 or transient elastography value of > 7 kPa or evidence of cirrhosis (F4) based on clinical criteria (or an APRI score of > 1 or transient elastography value of > 12.5 kPa), regardless of HBV DNA or ALT levels’
2	A 36 year old male patient is diagnosed with chronic hepatitis B. Laboratory testing shows HBV DNA levels of 3000 IU/mL and ALT levels above the upper limit of normal	According to the ‘Guidelines for the prevention, diagnosis, care and treatment for people with chronic hepatitis B infection’ by the WHO, should this patient be treated?	Treatment is recommended for all adults and adolescents (aged ≥ 12 years) with chronic hepatitis B […] with: HBV DNA > 2000 IU/mL (2015: > 20 000 IU/mL) ‘and an ALT level above the upper limit of normal (ULN) (30 U/L for men and boys and 19 U/L for women and girls)’
3	A 27 year old female patient is pregnant in the second trimester and tests positive for HBsAg. She lives in an area, in which HBV DNA and HBeAg testing is not available	What is the current recommendation regarding prevention of mother‐to‐child transmission of HBV according to the ‘Guidelines for the prevention, diagnosis, care and treatment for people with chronic hepatitis B infection’ by the WHO?	‘In settings where neither HBV DNA nor HBeAg testing is available, prophylaxis with tenofovir disoproxil fumarate may be considered for all HBV‐positive (HBsAg‐positive) pregnant women (preferably from the second trimester of pregnancy until at least delivery or completion of the infant HBV vaccination series), to prevent the mother‐to‐child transmission of HBV.’ (2015: no treatment recommendation in the above mentioned setting)
4	A 46 year old male patient is diagnosed with chronic hepatitis B. He has established osteoporosis and impaired kidney function	Which are the recommended treatment options for antiviral therapy according to the ‘Guidelines for the prevention, diagnosis, care and treatment for people with chronic hepatitis B infection’ by the WHO?	‘Entecavir or tenofovir alafenamide (if available) are recommended for people with established osteoporosis and/or impaired kidney function, […] as alternative regimens, for whom antiviral therapy is indicated.’ (2015: use entecavir or dose reduction of tenofovir)
5	A 52 year old female patient is diagnosed with chronic hepatitis B. To guide treatment eligibility and monitoring of the response, a HBV DNA assay is needed. However, a laboratory‐based HBV DNA assay is not available	Are there recommended alternatives according to ‘Guidelines for the prevention, diagnosis, care and treatment for people with chronic hepatitis B infection’ by the WHO?	‘Point‐of‐care HBV DNA nucleic acid test assays may be used as an alternative approach to laboratory‐based HBV DNA testing to assess HBV DNA level for treatment eligibility and to monitor treatment response. ‘(2015: Point‐of‐care HBV DNA nucleic acid test assays are listed in research gaps; no recommendation as alternative test)

*Note:* Five cases were invented to highlight the differences in treatment recommendations based on the new 2024 WHO guidelines compared to the ones from 2015.

### 2.2 GPT‐4 Prompting

GPT‐4 was accessed online (https://chat.openai.com/) on April 3rd and 4th, 2024, and operated as the February 13th 2024 version (GPT‐4 v0613). For each of the constructed cases, GPT‐4 was given the case vignette and a question of what the current treatment recommendations are according to the WHO guidelines for chronic hepatitis B. The prompting sequence was performed in a standardised format as follows:

Prompt: Case description + question.

Here, ‘case description’ and ‘question’ refer to Table 1, for example, the prompt for case 1 reads as follows:

‘A 14‐year‐old female patient is diagnosed with chronic hepatitis B by testing positive for HBsAg. The patient has no cirrhosis, but significant fibrosis diagnosed by transient elastography.

According to the ‘Guidelines for the prevention, diagnosis, care and treatment for people with chronic hepatitis B infection’ by the WHO, should this patient be treated?’

Each query was then repeated, but this time supplementing the prompt with the complete 2024 WHO guidelines as a *.pdf‐file in the chat interface using the ‘Attach file’ button simultaneously with the prompt. This way, GPT‐4 incorporates the retrieved information of the attached file into its answer. For each case, prompts were entered in a new chat interface, with the non‐contextualised prompt always preceding the contextualised one within the same session. For all cases, answers were recorded for analysis.

### 2.3 Evaluation of Treatment Recommendations

Two board‐certified internists (K.M.S., K.G.) and five residents in internal medicine (M.R.P., J.J., J.C., M.R.M., E.K.) evaluated the treatment recommendations proposed by GPT‐4 with and without context. On average, the readers had 4.6 ± 2.2 years of work experience (range from 8 months to 84 months) and have treated 118 ± 163 patients with CHB. Treatment recommendations provided by GPT‐4 were rated separately by every reader by answering the questions as shown in Table 2. The consistency of GPT‐4's answers with the new 2024 WHO guidelines was assessed in a binary manner (yes/no). Likert scales ranging from 1 to 3 were used to rate treatment recommendations regarding the inclusion of all relevant information given in the case vignette, the presence of textual errors as well as clear and precise wording. Textual errors were defined as seemingly correct responses that were nonsensical when considered against common knowledge in hepatology or as incorrect answers regarding the type of recommendation or level of evidence as specified in the guidelines. The average scores of the seven readers were normalised for all four questions and for both settings (with and without context), so that a score of 1 means ‘yes’ or ‘3’ and a score of 0 means ‘no’ or ‘1’ for the binary and Likert‐scale questions, respectively. Finally, the normalised average scores of all items were compared between GPT‐4's answers with and without context.

**TABLE 2:** Questions used to rate the provided treatment recommendations for each case.

Questions to evaluate	Possible answers
‘Are the treatment recommendations coherent with the 2024 WHO guidelines?’	‘Yes’–‘no’
‘Do the treatment recommendations consider the information given in the case description?’	Does not consider relevant case information Considers some, but not all, key case information Considers all relevant case information
‘Do the treatment recommendations contain textual errors?’	Contains multiple significant textual errors or nonsensical statements Contains minor textual errors Contains no textual errors
‘Are the treatment recommendations clearly and precisely worded?’	Wording is unclear, ambiguous, or imprecise Wording is somewhat clear but could be improved Wording is clear, precise, and unambiguous

*Note:* Seven physicians evaluated the answers given by GPT‐4 by using binary schemes and Likert scales (1 to 3).

The impact of contextualization was assessed using paired Wilcoxon signed‐rank tests to compare normalised rater‐specific average scores for each metric and normalised case‐specific combined scores (averaged across raters) between contextual conditions (α = 0.05). Inter‐rater reliability was evaluated using Gwet's first‐order agreement coefficient for categorial variables (guideline coherence) and intraclass correlation coefficient for continuous variables (all information considered, avoidance of textual errors, clear and precise wording). All analyses were conducted in R [17] (v4.4.2, R Foundation for Statistical Computing, Vienna, Austria).

## 3 Results

**FIGURE 1:**
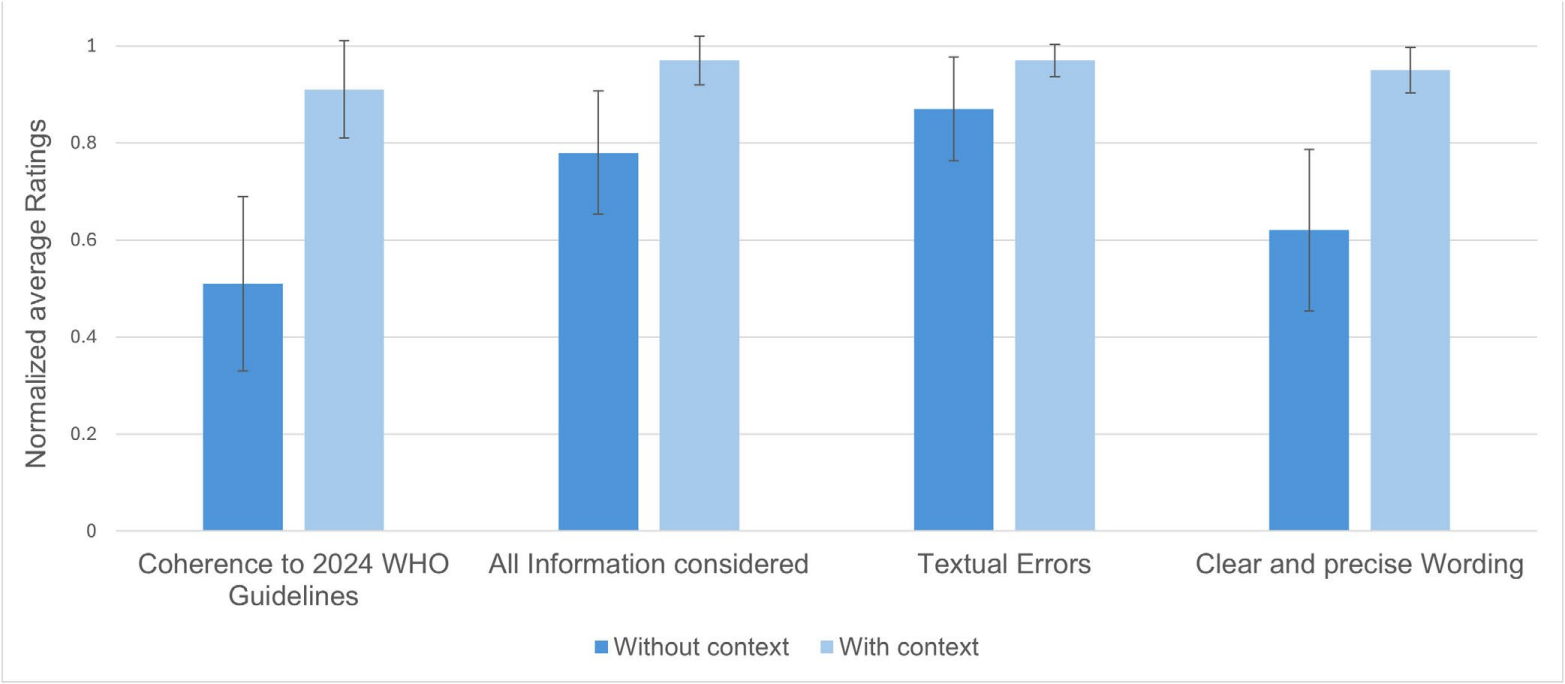
Multidimensional normalised average ratings of the treatment recommendations given by GPT‐4 with and without context as assessed by the seven physicians (error bars indicate the standard error of the mean ratings per category across all readers). Binary answers regarding coherence with guidelines were transformed into numerical values (1 equals ‘yes’ and 0 equals ‘no’). The remaining three categories were normalised to a scale from 0 (no agreement) to 1 (full agreement). Increasing the context by provision of external databases drastically improves guideline coherence and, to a lesser extent, also all remaining categories.

An overview of the average scores of the four questions with and without context is shown in Figure 1 and Table S1. The introduction of contextualisation improved each of the four items. Most importantly, guideline coherence, defined as the agreement of GPT‐4's treatment recommendations with the 2024 WHO guidelines, increased significantly: without context, only 51% of the cases were assessed as coherent on average, whereas with context, this rose to 91%, representing an increase of 78% (*p* = 0.017). The preciseness and clarity of wording in the recommendations also showed a substantial improvement, changing from an average score of 1.86 (where a score of 1 indicated unclear/imprecise and 3 indicated clear/precise) to 2.86 when contextualised (an increase of 54%, *p* = 0.011). The extent to which the recommendations considered all relevant information provided in the case vignette improved from an average score of 2.34 to 2.91 (where a score of 3 indicated all information was considered), leading to an increase of 24% (*p* = 0.011). Finally, textual correctness, assessing the absence of nonsensical statements or factual errors in the recommendations, was affected least, increasing from an average score of 2.60 to 2.91 (where a score of 3 indicated no textual errors) or by 12% (*p* = 0.029).

Without additional context, the mean combined normalised rating across cases was 0.70 (Table S2). With context, the mean combined normalised rating increased to 0.95, representing an average improvement of 38% (*p* = 0.031). Combined normalised ratings for the cases ranged from 0.62 to 0.77 without context and from 0.88 to 1.00 in the contextualised setting, reflecting only low heterogeneity among case ratings. The lowest combined normalised rating was recorded for case 3 in both configurations (0.62 without context and 0.88 with context), while the highest combined rating was observed for case 1 non‐contextualised (0.77) and for case 2 contextualised (1.00).

Inter‐rater reliability was fair for guideline coherence as expressed by a Gwet's first‐order agreement coefficient of 0.52. Agreement between raters regarding the Likert‐scale questions was good, based on an intraclass correlation coefficient of 0.80.

## 4 Discussion

This study evaluated the impact of contextualisation by provision of an auxiliary knowledge source for GPT‐4 in generating up‐to‐date treatment recommendations for CHB. Our findings showed that GPT‐4, when enhanced with more context, aligns consistently with the latest WHO guidelines, whereas GPT‐4 without additional context mostly adhered to the older 2015 WHO guidelines. Moreover, contextualisation improved the given treatment recommendations regarding clarity and preciseness and enhanced the amount of integration of all relevant information that was given in the case vignette.

The literature on the application of LLMs, particularly in medical contexts, is still evolving. In the past year, many studies indicated the high potential of LLMs in healthcare applications, ranging from automating routine tasks [18, 19] over patient education [20, 21] to support in clinical decision‐making [22, 23, 24]. Few studies have already evaluated the particular use case of providing LLM‐based treatment recommendations in accordance with specific guidelines, for example, in orthopedics [25], urology [26] and psychiatry [27]. While most studies confirm GPT‐4's usefulness and high potential in treatment guidance, there is consensus that LLMs, at least at the moment, occasionally provide incorrect information called ‘hallucinations', which may cause a spread of misinformation and, at worst, may put patients at risk due to overreliance [6]. Therefore, the clinical use of LLMs has to be accompanied by appropriate safeguarding measures to prevent patient harm.

Another method to increase LLM accuracy apart from prompt engineering with file attachment used in this study is retrieval augmented generation (RAG). In RAG, the LLM is connected to an external database that is often divided into manageable so‐called ‘chunks’ of information, allowing the model to retrieve only the most relevant sections in real time. This chunking approach ensures that RAG pulls precise, contextually relevant details from large datasets, in contrast to prompt engineering, which relies on attachment of pre‐selected sources to a chatbot interface. Only very few studies evaluated RAG‐enhanced LLMs in the context of medical treatment recommendations. In the context of gastrointestinal diseases, Lim et al. investigated this approach for appropriate colonoscopy intervals [28] and Kresevic et al. for the management of chronic Hepatitis C Virus infection [29], both of whom confirmed the beneficial effect of RAG regarding guideline coherence.

Therefore, a key difference of our study compared to the above‐mentioned studies is that we utilised the publicly available GPT‐4 chatbot interface, including its file attachment feature, whereas studies implementing RAG typically leverage API access and custom coding for more sophisticated, automated data retrieval. However, a key strength and distinct characteristic of this study lies in the close temporal proximity of LLM prompting and the release of the new CHB guidelines. By executing the study only a few days after the guideline release, it was highly unlikely that GPT‐4 had already been trained on these updated recommendations. Consequently, this setup allowed us to specifically isolate and demonstrate the impact of providing access to an up‐to‐date external document, effectively showcasing GPT‐4's vulnerability to providing outdated or incorrect treatment recommendations without immediate contextualisation. On top of that, our study did not solely assess the accuracy of the LLM output but also provided an analysis regarding clarity, completeness of incorporation of case vignette information, and presence of textual errors. Our findings align well with the current literature, supporting the beneficial role of providing external information sources when using LLMs in clinical decision‐making. However, treatment recommendations provided by LLMs may still contain content errors, especially in the context of rapidly evolving knowledge and guidelines derived therefrom, which highlights the necessity of critical validation of LLM‐based answers by healthcare professionals. Future work could focus on additional safeguarding measures such as a voting system by multiple LLM agents or an additional fact‐checking LLM that takes the provided output and checks for internal consistency.

While the findings of this study are promising, they come with several limitations that merit consideration. Firstly, the study's focus on a single disease (CHB) and its management according to one specific set of guidelines (WHO 2024) may limit the generalisability of the results. Furthermore, our sample size of cases was relatively small, which may affect the robustness and statistical power of our conclusions. The choice of sample size was guided by the changes in the new guidelines. Cases in which both guidelines agree do not allow for differentiation of which information GPT‐4 had been accessing. Additionally, our study did not explore the impact of conflicting information sources or guidelines that might result in erroneous retrievals, thus potentially overstating its efficacy. Another limitation lies in the method of information retrieval in this study: our use of the chatbot interface rather than a more controlled API‐based RAG setup introduces variability inherent to the platform's retrieval mechanism when processing attached files, which may differ from optimised RAG systems. Furthermore, retention bias cannot be fully excluded, as both the non‐contextualised and contextualised prompts were submitted within the same chat session. Although the non‐contextualised prompt always preceded the contextualised one, GPT‐4 may have retained internal representations that influenced the subsequent response in the contextualised condition. Finally, GPT‐4 has been shown to perform poorly regarding information extraction of graphical tables or flowcharts [30], which may have compromised the treatment recommendations given by the LLM. Future research should aim to address these limitations by expanding the scope of diseases, guidelines, and sample sizes tested, thereby providing a more comprehensive understanding of the utility and limitations of contextualisation when using LLMs in clinical practice. Such efforts are essential for harnessing the full potential of advanced AI tools in improving healthcare outcomes through accurate, reliable, and up‐to‐date medical recommendations.

## 5 Conclusion

Increasing the provided context by attachment of external data sources may significantly enhance the capabilities of GPT‐4, particularly in the context of generating treatment recommendations that are coherent with current medical guidelines. However, scattered misinformation may still occur, necessitating validation of LLM‐based treatment guidance by healthcare professionals.

## Author Contributions

R.S., C.V.S., S.N., C.K., J.N.K. and D.T. designed the study. C.V.S. created the five cases. J.N.K. and K.G. reviewed the cases. R.S., M.S.S. and I.A. queried GPT‐4 and prepared the reader study. K.G., K.M.S., M.R.P., M.M., J.J., E.K., M.R.M. and J.C. performed the reader study. R.S. and D.T. analysed the results of the reader study. R.S. wrote the manuscript. All authors read, corrected and approved the manuscript.

## Ethics Statement

The study was conducted in accordance with the ethical principles outlined in the Declaration of Helsinki. As this study did not involve human subjects or animal experiments, no ethical approval was required.

## Conflicts of Interest

J.N.K. declares consulting services for Owkin, France, and Panakeia, UK, and has received honoraria for lectures by Bayer, Eisai, MSD, BMS, Roche, Pfizer, and Fresenius. J.N.K. and D.T. hold shares in StratifAI GmbH and Synagen GmbH, Germany. D.T. has received honoraria for lectures by Bayer and Philips. No other potential conflicts of interest are declared by any of the authors.

## Supporting information


**TABLE S1:** The average ratings of treatment recommendations provided by GPT‐4, with and without contextualization, were evaluated across multiple dimensions by seven physicians. Answers regarding guideline coherence were binary (0: no; 1: yes) while answers to the other items were provided on a Likert‐scale from 1 to 3. The incorporation of the current guideline file in the prompt significantly enhances guideline coherence and, to a lesser degree, improves performance in the remaining categories as well.
**TABLE S2:** Average combined normalised ratings of treatment recommendations provided by GPT‐4 per case, with and without contextualization, as evaluated by the seven physicians.

## Data Availability

All data needed to reproduce the study can be found in the manuscript except the WHO guidelines which are publicly accessible (2015: https://www.who.int/publications/i/item/9789241549059; 2024: https://www.who.int/publications/i/item/9789240090903).
