# Supplementary material for: The Impact of Access to Clinical Guidelines on LLM‐Based Treatment Recommendations for Chronic Hepatitis B
Source: Liver Int. 2025 Sep 2;45(10):e70324. doi: 10.1111/liv.70324 (PMC12402858; doi:10.1111/liv.70324)
Supplement: Supplementary file 1 — TABLE S1: The average ratings of treatment recommendations provided by GPT‐4, with and without contextualization, were evaluated across multiple dimensions by seven physicians. Answers regarding guideline coherence were binary (0: no; 1: yes) while answers to the other items were provided on a Likert‐scale from 1 to 3. The incorporation of the current guideline file in the prompt significantly enhances guideline coherence and, to a lesser degree, improves performance in the remaining categories as well. TABLE S2: Average combined normalised ratings of treatment recommendations provided by GPT‐4 per case, with and without contextualization, as evaluated by the seven physicians. [file LIV-45-0-s001.docx]

| **Items** | **Mean ratings** | |
| --- | --- | --- |
|  | **Without context** | **With context** |
| Coherence with 2024 WHO Guidelines | 0.51 ± 0.18 | 0.91 ± 0.10 |
| All Information considered | 2.34 ± 0.38 | 2.91 ± 0.15 |
| Avoidance of Textual errors | 2.60 ± 0.32 | 2.91 ± 0.10 |
| Clear and precise Wording | 1.86 ± 0.50 | 2.86 ± 0.14 |

**Supplementary Table 1.** The average ratings of treatment recommendations provided by GPT-4, with and without contextualization, were evaluated across multiple dimensions by seven physicians. Answers regarding guideline coherence were binary (0: no; 1: yes) while answers to the other items were provided on a Likert-scale from 1 to 3. The incorporation of the current guideline file in the prompt significantly enhances guideline coherence and, to a lesser degree, improves performance in the remaining categories as well.

| **Cases** | **Mean combined ratings** | |
| --- | --- | --- |
|  | **Without context** | **With context** |
| Case 1 | 0.77 ± 0.11 | 0.99 ± 0.02 |
| Case 2 | 0.76 ± 0.15 | 1.0 ± 0.0 |
| Case 3 | 0.62 ± 0.22 | 0.88 ± 0.10 |
| Case 4 | 0.68 ± 0.12 | 0.96 ± 0.04 |
| Case 5 | 0.64 ± 0.30 | 0.93 ± 0.04 |

**Supplementary Table 2.** Average combined normalized ratings of treatment recommendations provided by GPT-4 per case, with and without contextualization, as evaluated by the seven physicians.
